# The role of generative adversarial networks in brain MRI: a scoping review

**DOI:** 10.1186/s13244-022-01237-0

**Published:** 2022-06-04

**Authors:** Hazrat Ali, Md. Rafiul Biswas, Farida Mohsen, Uzair Shah, Asma Alamgir, Osama Mousa, Zubair Shah

**Affiliations:** College of Science and Engineering, Hamad Bin Khalifa University, Qatar Foundation, 34110 Doha, Qatar

**Keywords:** Artificial intelligence, Data augmentation, Generative adversarial networks, Magnetic resonance imaging, Medical imaging

## Abstract

The performance of artificial intelligence (AI) for brain MRI can improve if enough data are made available. Generative adversarial networks (GANs) showed a lot of potential to generate synthetic MRI data that can capture the distribution of real MRI. Besides, GANs are also popular for segmentation, noise removal, and super-resolution of brain MRI images. This scoping review aims to explore how GANs methods are being used on brain MRI data, as reported in the literature. The review describes the different applications of GANs for brain MRI, presents the most commonly used GANs architectures, and summarizes the publicly available brain MRI datasets for advancing the research and development of GANs-based approaches. This review followed the guidelines of PRISMA-ScR to perform the study search and selection. The search was conducted on five popular scientific databases. The screening and selection of studies were performed by two independent reviewers, followed by validation by a third reviewer. Finally, the data were synthesized using a narrative approach. This review included 139 studies out of 789 search results. The most common use case of GANs was the synthesis of brain MRI images for data augmentation. GANs were also used to segment brain tumors and translate healthy images to diseased images or CT to MRI and vice versa. The included studies showed that GANs could enhance the performance of AI methods used on brain MRI imaging data. However, more efforts are needed to transform the GANs-based methods in clinical applications.

**Supplementary Information:**

The online version contains supplementary material available at 10.1186/s13244-022-01237-0.

## Key points

- This article aims to provide a comprehensive review on the applications of generative adversarial networks (GANs) in brain MRI.
- The specific focus of this education review is on brain MRI.
- It covers a large number of studies on GANs in brain MRI and the most recently published studies on brain MRI.

## Introduction

Magnetic resonance imaging (MRI) is a widely used medical imaging technology. MRI is non-intrusive and considered safe for humans. MRI can generate different modalities of an image and can provide valuable insights into a specific disease. The frequent sequences of MRI are T1-weighted and T2-weighted scans [1, 2]. The major difference between MRI and other medical imaging technologies is that MRI is free from using X-ray radiography. The radiologists use MRI to analyze brain tissue and diagnose brain-related diseases such as brain tumors (i.e., the abnormal and uncontrolled growth of brain cells). This process requires trained radiologists, and the accuracy is heavily dependent on the expertise of the radiologists and the quality of MRI data acquisition [1, 2].

Computer-aided diagnosis (CAD) can aid in the process of MRI analysis. Recently, there has been a significant increase in interest in developing artificial intelligence and deep learning-based methods for CAD. However, deep learning methods rely on training using large medical imaging data. Generative adversarial networks (GANs) have the potential to generate new samples of the data and represent the distribution of the real data. GANs are particular types of deep learning models formed of two neural networks, namely the generator and the discriminator. The generator generates new samples, while the discriminator attempts to classify the images as real or synthetic. The adversarial training effectively improves the overall training of the model. While GANs methods were initially popular for generating synthetic data in the medical imaging domain, they have also been used for other applications such as super-resolution, segmentation, and diagnosis.

This study performed a scoping review to find out the role of GANs-based methods in brain MRI. While many reviews have been performed on the use of GANs in medical imaging and GANs in MRI [1–3], their scope is too broad. For example, the review in [1] covers a broad range of MRI and does not focus on brain MRI only. Similarly, the review in [2] covers many different deep learning techniques and does not limit the discussion to GANs-based methods only. The review in [3] covers the discussion on GANs for all types of medical imaging data. Table 1 provides a comparison of our work with previous reviews. The growing number of studies on the use of GANs in brain MRI demands a dedicated review. In this regard, this review presents a review of how GANs-based methods were used to address many challenges in advancing the performance of AI for brain MRI data. More specifically, it summarizes the applications of GANs-based methods in brain MRI such as synthesis of brain MRI, segmentation of brain tumor, and super-resolution of brain MRI. Furthermore, it also highlights the different evaluation metrics such as structural similarity index measure (SSIM) and the peak signal-to-noise ratio (PSNR) used in the literature for evaluation of the performance of GANs. The following research questions related to the role of GANs-based method in brain MRI were considered for this review.

1. What were the typical applications of GANs proposed for brain MRI?
2. Which architectures of GANs are most commonly applied for brain MRI?
3. What was the purpose of using GAN in brain MRI?
4. What were the most commonly used datasets for brain MRI?
5. How many datasets were publicly accessible?
6. What evaluation matrices were used for the validation of the model?

**Table 1:** Comparison with previous reviews

Previous review	Year	Scope and coverage	Comparative contribution of our review
An overview of deep learning in medical imaging focusing on MRI [1]	2019	(1) It did not focus on GANs but rather covered many different deep learning methods (2) It did not focus on just brain MRI but rather focused on different MRI (3) It did not cover many recent studies as there has been an exponential rise in GANs-based methods for brain MRI during the last 2 years	(1) Our review is focused on GANs (2) Our review is focused on brain MRI (3) Our review covers many recent studies, published in 2020 and 2021
Review of deep learning approaches for the segmentation of multiple sclerosis lesions on brain MRI [2]	2020	(1) It did not focus on GANs but rather covered a broad range of deep learning methods (2) It did not cover applications for brain MRI such as synthesis of brain MRI data, translation of brain MRI data, and deep learning for noise removal from brain MRI, etc.	(1) Our review is focused on GANs (2) Our review covers all the possible applications for brain MRI
Generative adversarial network in medical imaging: A review [3]	2019	(1) It did not focus on brain MRI but rather covered all modalities of medical imaging (2) It did not cover many recent studies published in 2020 and 2021, as there has been an exponential rise in studies for brain MRI during the last 2 years	(1) Our review is focused on brain MRI (2) Our review covers many recent studies, published in 2020 and 2021

The study will be helpful for researchers and professionals in the medical imaging and healthcare domain who are considering using GANs methods to diagnose and predict the brain tumors from the MRI images. The review also lists publicly available brain MRI datasets that will be helpful for AI researchers to develop advanced research methods.

## Methods

We performed a literature search in famous databases and conducted a scoping review as per the guidelines of the PRISMA-ScR (Preferred Reporting Items for Systematic Reviews and Meta-Analyses Extension for Scoping Reviews) [4]. Additional file 1: Table S1 provides the adherence to the PRISMA-ScR checklist. The following methods were used for the search and the study selection.

### Search strategy

#### Search sources

This review searched five different databases for relevant literature, namely PubMed, Scopus, IEEE Xplore, ACM Digital Library, and Google Scholar. We note here that MEDLINE is covered in PubMed. The search was performed between September 20 and 22, 2021. For the search outcomes of Google Scholar, only the first 100 results were considered, as, beyond the first 100 entries, the search results were quickly losing match and relevancy to the topic of the review. In addition to the search on the five databases, we also screened the reference lists of the included studies to find additional relevant studies.

#### Search terms

We defined the search terms from the available literature and by referring to the experts in the fields. The search terms were selected based on the intervention (e.g., deep learning, generative adversarial networks (GANs)), the target anatomy (brain), and the target data modality (e.g., MRI, fMRI, sMRI). The search strings used in this study are provided in Additional file 1: Table S2.

### Search eligibility criteria

We focused on GANs-based approaches used for brain MRI data. We considered studies published in English from January 2015 to September 2021. Studies for all applications of GANs were included, such as segmentation, synthesis, noise removal, and super-resolution of brain MRI. We included studies that used GANs for brain MRI data and excluded studies that used other deep learning methods (such as convolutional neural networks or recurrent neural networks) but did not use GANs. Similarly, we excluded studies that used GANs for non-image data or image data of modalities other than MRI (such as ultrasound, X-ray, or computed tomography (CT)). We also excluded studies that used GANs for MRI data other than the brain.

We included peer-reviewed articles and conference proceedings and excluded preprints, commentaries, short reviews, editorials, and abstracts. Similarly, we excluded studies that presented a survey of GANs methods. No restrictions were imposed on the country of publication, comparators, and outcomes of the GANs methods.

### Study selection

Two reviewers, namely authors AJ and OT, independently reviewed the titles and abstracts of the studies identified in the search and made initial flagging for inclusion and exclusion. The flagging was then verified by a third reviewer (HA). The studies that passed the title and abstract screening were shortlisted for the full-text reading phase to perform study selection. Any disagreement between the reviewers (AJ and OT) was investigated and resolved through discussion and consensus. The Cohen’s kappa score [5] was calculated to measure the agreement between the two reviewers.

### Data extraction

We prepared a purpose-built form for data extraction. Additional file 1: Table S4 shows the data extraction form. The entries for the form were pilot-tested using ten relevant studies to extract the data accurately. Two reviewers (MB and FA) independently performed the data extraction according to the data extraction form. The data were extracted for the applications of the studies, the purpose of using GAN, the type of GAN, features of the dataset, and the evaluation mechanism of the GANs-based methods. Any disagreement between the two reviewers was resolved through discussion and consensus.

### Data synthesis

After the extraction of the data from the included studies, we synthesized the data using a narrative approach. First, we classified the included studies in terms of their applications, such as synthesis (data augmentation), diagnosis (e.g., tumor detection), prognosis, and super-resolution. We also classified the studies based on the purpose of using GANs, such as synthesis, noise removal, and translation. Based on dataset types, we organized the data into two broad categories: studies that used publicly available datasets and studies that used privately collected MRI data. We also summarized the studies based on the size of the dataset, the evaluation mechanisms, and the reporting of external validation. We performed and managed the data synthesis using MS Excel.

## Results

### Search and study selection results

**Fig. 1:**
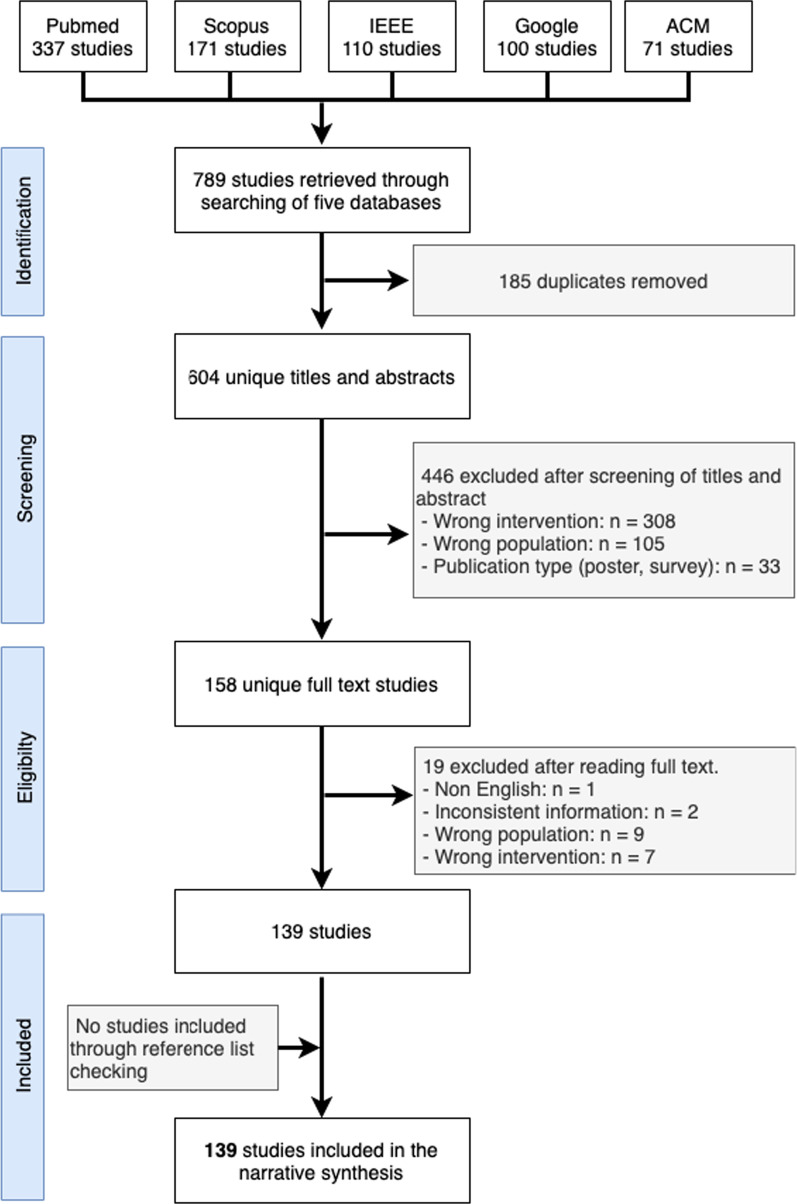
The PRISMA-ScR flowchart for the selection of the included studies

We retrieved 789 studies as a search result. We removed 185 duplicates. We then did the screening of the titles and abstracts of the remaining studies. As a result of title and abstract screening, we excluded 446 studies following the criteria defined in the protocol. We then performed the full-text reading of the remaining 158 studies. Among these, we removed 19 studies that did not fulfill the criteria of inclusion. Finally, we were left with 139 studies for inclusion in this survey. See Fig. 1 for the flowchart of the study selection process. No additional studies were identified by forward-and-backward reference checking. The Cohen’s kappa score was 86.3% for the title and abstract screening, which shows a good agreement between the reviewers. The Cohen’s kappa score was 84.7% for the full-text reading phase, which shows a good agreement between the reviewers. Additional file 1: Table S3 shows the matrix for the calculation of the Cohen’s kappa score.

### Demographics of the included studies

**Fig. 2:**
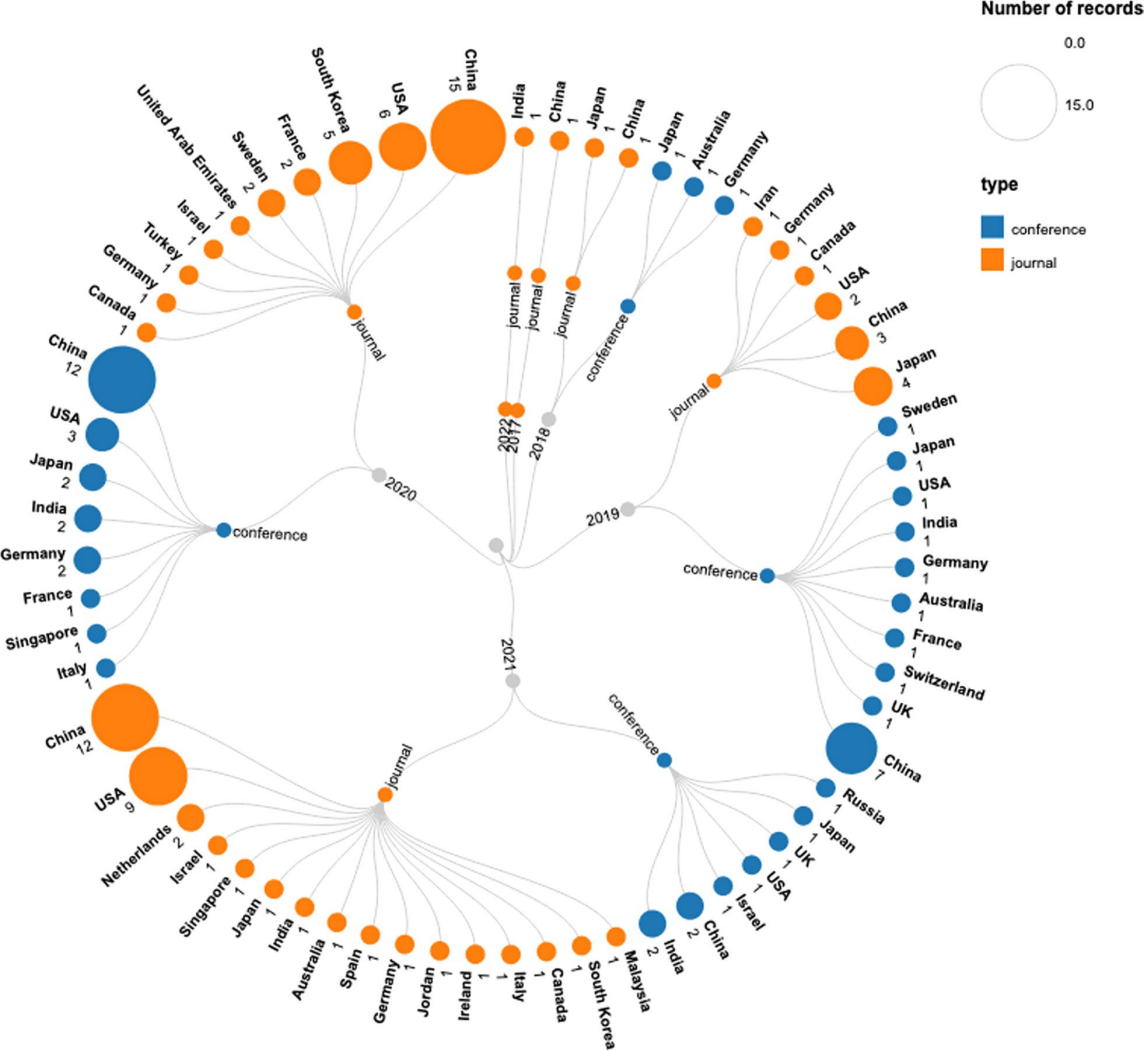
Year-wise and country-wise distribution of the included studies. The numbers at the terminal node show the number of publications in each country

Among the included studies, 87 were peer-reviewed journal articles and 52 were conference publications. More than two-thirds of the studies (*n* = 104) were published in the last 2 years, i.e., 2020 and 2021. In comparison, only five studies were published in 2018 and only one study was published in 2017. A total of 27 countries contributed to the studies. Around one-third of the studies (*n* = 53) were published in China. The only two other countries that published more than ten studies were the USA (*n* = 21) and Japan (*n* = 12). Table 2 summarizes the demographics of the included studies. Figure 2 shows a visualization of the year-wise and country-wise distribution of the included studies.

**Table 2:** Demographics of the included studies

	Number of studies
*Year*	
Year	
2022	1
2021	44
2020	60
2019	28
2018	5
2017	1
*Countries*	
Country	
China	53
USA	22
Japan	11
Germany	7
India	7
South Korea	6
France	4
Sweden	3
Israel	3
Canada	3
Australia	2
UK	2
Singapore	2
The Netherlands	2
Italy	2
United Arab Emirates	1
Turkey	1
Switzerland	1
Spain	1
Russia	1
Malaysia	1
Jordan	1
Ireland	1
Iran	1
Malaysia	1
*Type of publication*	
Venue	
Conference	52
Journal	87

### Applications of GANs in brain MRI

**Fig. 3:**
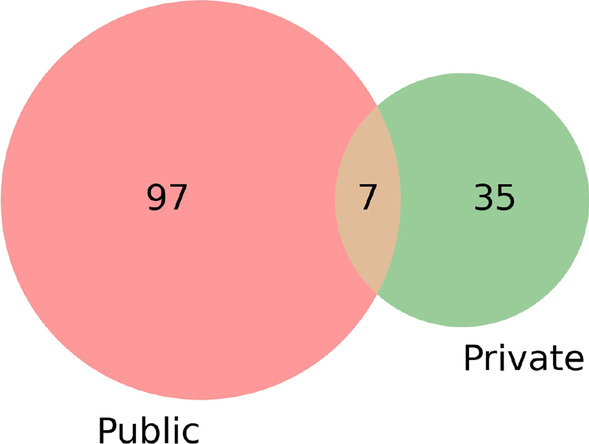
Venn diagram for the number of studies using public vs. privately collected datasets. Some of the studies (*n* = 7) reported using both public and private datasets

GANs have been used for many applications of brain MRI data. The included studies used GAN-based methods as a sub-module of their deep learning frameworks for different applications, as shown in Table 2. The majority of the included studies targeted applications, namely the generation of synthetic data (*n* = 43), the segmentation of area of interest in brain MRI (*n* = 32), and the diagnosis of neurological diseases (*n* = 22). Other common applications of the studies were super-resolution to improve the quality of the images as reported in ten studies and reconstruction of high-quality images (which can be considered a sub-category of super-resolution) reported in 13 studies. Few studies also reported applications such as noise removal (*n* = 5), prognosis (*n* = 4), and image registration (*n* = 2). Only one study reported the generation of 3D synthetic volumes of MRI data (see Fig. 3).

The included studies used GANs for many different applications, namely synthesis (generation of synthetic data), segmentation (generation of the segmentation mask), diagnosis, and translation of data from one modality to another (e.g., translation from CT to MRI and vice versa, or translation form normal MRI to infected MRI). Almost one-third of the studies (*n* = 45) reported the use of GANs for the synthesis of data. Around one-sixth of the studies (*n* = 26) reported GANs to perform segmentation. Other popular use cases of GANs were diagnosis reported in 16 studies, reconstruction reported in 15 studies, and translation reported in 12 studies. The reconstruction may also be regarded as a particular case of image synthesis. Only a few studies reported use cases of GANs for other applications, such as super-resolution reported in seven studies, noise removal reported in five studies, prediction reported in five studies, and prognosis reported in four studies. Table 3 provides a summary of the use cases of GANs.

**Table 3:** Applications of the use of GANs in brain MRI

Applications of studies	No. of studies	Reference of the study
*Applications of studies*		
Synthesis	43	[6–37, 39–49]
Segmentation	32	[50–81]
Diagnosis	22	[82–103]
Reconstruction	13	[119–131]
Super-resolution	10	[104–112, 118]
Prediction	7	[132–138]
Noise removal	5	[113–117]
Prognosis	4	[139–142]
Image registration	2	[143, 144]
3D synthesis	1	[38]
*Purpose of using GANs*		
Synthesis	45	[6–35, 53–59, 96–100, 120, 132, 133]
Segmentation	26	[60–81, 101, 102, 104, 104]
Diagnosis	16	[50–52, 82–93, 106]
Reconstruction	15	[12–123, 125–131]
Translation	12	[37, 41–49, 95, 118, 143]
Super-resolution	7	[38, 107–112]
Noise removal	5	[113–117]
Prediction	5	[134–138]
Prognosis	4	[139–142]
Features extraction	1	[39]
Translation	1	[37, 41–45, 47–49, 95, 118, 143]
Anomaly detection	1	[94]
Image registration	1	[144]

### Types of GANs methods

While there are many different types of GANs usually named based on their architectures, there is a tendency to assign a new name to every GAN even if the fundamental changes in the architecture are not significant. This review found that the most common types of GANs used were the cycleGAN used by 12 studies [15, 17, 48, 51, 55–57, 65, 66, 79, 84, 110, 133] followed by conditional GAN used by 8 studies [53, 54, 71, 72, 101, 112, 118, 119], and Wasserstein GAN used by 7 studies [13, 14, 19, 39, 116, 131, 132]. Other types of GANs reported in more than one study were deep convolutional GAN, reported in three studies [20, 93, 140], unified GAN [21, 49] reported in two studies, and Pix2Pix GAN, reported in two studies [32, 133].

### Types of datasets

Most of the studies (*n* = 97) reported the use of public datasets for brain MRI for the training of GAN models. Thirty-five studies reported the use of privately collected data. A few studies (*n* = 7) reported using both public and privately collected data. This review identified many different datasets used in the included studies. Table 4 provides a list of publicly available datasets and the access link. In the included studies, the most commonly used dataset was the *Alzheimer’s Disease Neuroimaging Initiative* dataset reported in 16 studies (also see Table 4). The BRaTs 2018 dataset was reported in eight studies, while the use of the IXI dataset of MR images from three different hospitals in London was reported in seven studies. The accumulative number of studies using the various versions of the BRaTs dataset was 20.

**Table 4:** Publicly available datasets used in the included studies. Sorting is done on the basis of the number of studies using the dataset

Dataset name	URL	No. of studies	IDs of studies
Alzheimer’s Disease Neuroimaging Initiative (ADNI)	http://adni.loni.usc.edu/	16	[19, 27, 42, 51, 65, 69, 73, 84, 85, 87, 92, 95, 96, 139, 140, 143]
BRATS2018	https://www.med.upenn.edu/sbia/brats2018/data.html	8	[8, 10, 11, 22, 55, 56, 58, 78]
IXI dataset	http://brain-development.org/ixi-dataset/	7	[9, 13, 86, 106, 108, 110, 116]
BRATS2016	https://sites.google.com/site/braintumorsegmentation/home/brats_2016	4	[6, 7, 14, 50]
Connectome	https://sites.google.com/view/calgary-campinas-dataset/home	3	[36, 123, 128]
BrainWeb	https://brainweb.bic.mni.mcgill.ca/	3	[47, 113, 116]
Decathlon	http://medicaldecathlon.com/	3	[52, 63, 77]
Figshare	https://figshare.com/articles/dataset/brain_tumor_dataset/1512427	3	[35, 90, 103]
	http://www.developingconnectome.org	3	[104, 105, 107]
BRATS 2013	https://www.smir.ch/BRATS/Start2013	2	[21, 91]
BraTS 2015	https://sites.google.com/site/braintumorsegmentation/home/brats2015	2	[16, 53]
BraTS 2017	https://www.med.upenn.edu/sbia/brats2017/data.html	2	[71, 98]
HCP	https://www.humanconnectome.org/study/hcp-young-adult	2	[12, 110]
Cancer Imaging	https://wiki.cancerimagingarchive.net/pages/viewpage.action?pageId=24282666	2	[37, 83]
PPMI	www.ppmi-info.org/data	2	[39, 97]
	http://epipage2.inserm.fr	2	[105, 107]
Brats 2014	https://www.virtualskeleton.ch/BRATS/Start2014	1	[142]
Brats 2019	https://www.med.upenn.edu/cbica/brats2019/data.html	1	[76]
ISLES	http://www.isles-challenge.org/ISLES2015/	1	[8]
NAMIC dataset	http://hdl.handle.net/1926/1687	1	[9]
MIT	http://twinsetfusion.csail.mit.edu/	1	[23]
MRIdata	http://mridata.org/	1	[36]
Harvard	http://www.med.harvard.edu/aanlib	1	[82]
VIM	http://crcns.org/data-sets/vc/vim-1	1	[40]
BIT China	https://isip.bit.edu.cn/	1	[60]
CIND	https://cind.ucsf.edu/	1	[80]
IBSR	https://www.nitrc.org/projects/ibsr	1	[113]
Hisub	http://www.nitrc.org/projects/mni-hisub25	1	[25]
ATAG	https://www.nitrc.org/projects/atag_mri_scans/	1	[115]
Cabal	https://github.com/cabal-cmu/Feedback-Discovery	1	[135]
John Hopkins University	http://iacl.ece.jhu.edu/index.php/MSChallenge	1	[125]
CSIRO	https://aibl.csiro.au/	1	[132]
NIFD	http://memory.ucsf.edu/research/studies/nifd	1	[6]
GDC	https://portal.gdc.cancer.gov/	1	[98]
UK Data Service	https://reshare.ukdataservice.ac.uk/851861/	1	[102]
NFB	http://preprocessed-connectomes-project.org/NFB_skullstripped/	1	[102]
ISEG2017	https://iseg2017.web.unc.edu/	1	[113]
OpenNeuro	https://openneuro.org/datasets/ds001506	1	[127]
ATLAS dataset	http://fcon_1000.projects.nitrc.org/indi/retro/atlas.html	1	[54]
OpenNeuro2	https://openneuro.org/datasets/ds001246/	1	[122]

The names of the dataset are assigned only for identification purposes and do not follow any specific convention

### Evaluation procedure

The number of patients was reported in some studies, while other studies reported the number of images. The maximum number of patients for whom the data were used was 2175 [92]. Two studies reported the use of more than 100,000 thousand images [23, 106], and one study reported the use of more than 10,000 images. In 25 studies, the number of images used was between 1000 and 10,000. In 33 studies, the number of images used was between 100 and 1000. Other studies either used less than 100 images or did not include information on the number of images. In the included studies, 38 reported splitting the data into independent training and test sets, while 17 reported splitting the data into training, validation, and test sets. Many other studies used the k-fold cross-validation method for evaluation; for example, twofold cross-validation was reported in three studies and sevenfold cross-validation was reported in two studies (see Table 5). External evaluation by human experts was reported in seven studies only.

**Table 5:** Evaluation mechanisms used in different studies

Evaluation mechanism	Number of studies	IDs of studies
Train, validate, test split	17	[6, 16, 17, 22, 37, 58, 59, 65, 76, 81, 89, 97–99, 106, 121, 126]
Training, test split	38	[10, 11, 13, 14, 20, 24, 33, 35, 36, 40, 45, 47, 50, 52, 53, 57, 66, 68, 69, 77, 87, 92, 98, 100, 103, 104, 107, 108, 110, 112, 115–117, 122, 125, 127, 128, 130]
Twofold cross-validation	3	[9, 75, 114]
Threefold cross-validation	2	[134, 137]
Fourfold cross-validation	2	[56, 70]
Fivefold cross-validation method	12	[7, 8, 21, 25, 29, 41, 46, 62, 90, 113, 120, 129]
Sevenfold cross-validation	2	[79, 139]
Tenfold cross-validation	6	[42, 80, 84, 95, 96, 101]
External	7	[31, 32, 43, 45, 48, 118, 135]

The different metrics used for the evaluation of the quality of the generated images using GANs were SSIM (*n* = 53 studies), PSNR (*n* = 49 studies), and FID (*n* = 8 studies). Other metrics for evaluation of performance for diagnosis, segmentation, or classification were Dice score used in 31 studies, mean absolute error used in 16 studies, and mean square error used in 16 studies. Table 6 summarizes the different evaluation metrics used in the studies.

**Table 6:** Most popular evaluation metrics used in different studies

Evaluation metric	Number of studies	IDs of studies
SSIM	53	[7–12, 15, 16, 18, 21, 25, 27, 36, 40, 42, 43, 45, 47, 48, 55, 56, 58, 62, 66, 69, 72, 85, 86, 103–110, 112, 113, 115–117, 120–123, 125–131]
PSNR	49	[7–11, 15–17, 21, 25, 36, 38, 42, 43, 45–48, 53, 55, 56, 58, 62, 66, 72, 85, 86, 97, 104–110, 112, 113, 115–118, 120, 121, 123, 124, 128, 129, 131]
DSC	31	[9, 20, 29, 45, 50–56, 59–61, 68, 72–77, 79–81, 102, 105, 114, 125, 136, 142–144]
Accuracy	22	[6, 13, 14, 19, 34, 35, 37, 39, 64, 83, 84, 89, 90, 92, 93, 95, 96, 98, 122, 132, 135, 139]
MAE	16	[7, 17, 21, 23, 29, 42, 46, 53, 58, 69, 85, 100, 115, 120, 129, 134]
MSE	16	[11, 16, 40, 45, 48, 58, 72, 117, 118, 122, 123, 128, 130, 131, 142]
Sensitivity	11	[75, 76, 81, 84, 92, 95, 96, 98, 99, 142]
Precision	9	[19, 26, 54, 64, 75, 132, 135, 138, 139]
Recall	9	[19, 26, 39, 54, 64, 132, 135, 138, 139]
F1 score	8	[19, 24, 64, 92, 93, 135, 138, 139]
FID	8	[21, 22, 42, 59, 109, 130]
Specificity	8	[68, 76, 84, 92, 95, 98, 142]

The numbers do not sum up as many studies used more than one evaluation metric, while some studies lack details on evaluation metrics

*SSIM* structural similarity index measure, *PSNR* peak signal-to-noise ratio, *DSC* Dice similarity coefficient, *MAE* mean absolute error, *MSE* mean square error, *FID* Frechet inception distance

### Focal diseases in the studies

We also identify the diseases that were the focus of the included studies. In the included studies, 44 studies reported their methods for addressing challenges related to brain tumors, such as tumor segmentation, tumor classification, or tumor growth prediction. Similarly, 20 studies reported the use of their methods for diagnosis, prognosis, or analysis of neurodegenerative disorders, for example, Alzheimer's disease, autism spectrum disorder (ASD), multiple sclerosis, and Parkinson’s disease. The remaining 75 studies did not focus on a particular disease. A summary of the disease-based categorization of the studies is given in Table 7.

**Table 7:** Focal diseases in the studies

Focal disease	Number of studies (n)	IDs of studies
Brain tumor	44	[5, 10, 20], [22], [25, 32, 35, 37, 44, 50–58, 61–64, 66–69, 71, 74–78, 83, 89, 90, 93, 98–101, 107, 133, 136, 142]
Neurodegenerative disorders	20	[19, 26, 31, 33, 39, 45, 84, 85, 87, 88, 91, 92, 94–97, 132, 137, 139, 140]
None	75	[7–9, 11–18, 21, 23, 24, 27–29, 31, 34, 36, 38, 40–43, 46–49, 59, 60, 65, 70, 72, 73, 79–82, 86, 102–106, 108–131, 134, 135, 138, 141, 143, 144]

## Discussion

### Principal results

In this study, we conducted a scoping review of the use of GANs in brain MRI data. We found that most of the studies were published in the years 2020 and 2021, while very few (only six) were published in 2016 and 2017 combined. This is not surprising as the interest in using GANs for medical imaging in general and brain MRI, in particular, gained momentum only recently. More than one-third of the studies were published in China (*n* = 53). The second-largest number of studies were published in the USA (*n* = 21), although less than half of those published in China. In comparison, only seven studies were published in India and Germany each. The rest of the countries published less than five studies each.

In almost one-third of the studies, the main application of using GANs was the synthesis/generation of data to achieve data augmentation. However, many studies also used GANs for the segmentation of tissues of interest, for example, the segmentation of tumors in brain MRI. Another popular use case of GAN was translating images from one modality to another or translating from normal to cancerous images. Furthermore, GANs can enhance the quality of images and hence were used for super-resolution of images as reported in seven studies and noise removal as reported in five studies. Less common use cases of GANs on brain MRI data were prognosis and image registration reported only in 4 studies and 1 study, respectively. While GANs are more popular for data synthesis, addressing a particular clinical disease is usually not the focus of using GANs. Nevertheless, some studies have demonstrated the effectiveness of GANs by demonstrating the use of the generated data to improve the diagnosis or prognosis of different diseases.

The term synthesis in this review is used in a broader sense and covers the synthesis of brain MRI sequences as well as the synthesis of missing sequences from existing sequences. The synthesized data were then used to enhance the diagnosis, such as detecting Alzheimer’s disease or segmentation of brain tumors.

The cycleGAN architecture that uses two GANs for generating synthetic data was the most popular choice of architecture in the included studies. Other popular choices were the Wasserstein GAN and the deep convolutional GAN. For many studies, the fundamental changes in the architecture were only minor, or the details on the changes introduced were insufficient; it is beyond the scope of this review to analyze all the architectures.

While testing the models on individual test sets or using k-fold cross-validation methods was reported in most of the studies, external validation of the performance is still limited and should be encouraged in future work.

### Research and practical implications

The majority of the included studies reported results on publicly available datasets. Among these, the BRaTS dataset and the Alzheimer’s Disease Neuroimaging Initiative dataset were the most popular datasets among the researchers. Since these datasets can be accessed publicly, it would be of great help to provide the associated computer code/software for the results reported in the included studies. This would encourage other researchers to reproduce the results and build upon the existing models/methods. However, some studies reported results on privately collected data. Hence, the opportunity for external validation of the claims made in the research studies or building upon those results is limited.

We did not find any framework implemented on mobile devices in the included studies. The computational requirements of GANs and the memory resources for MRI data can be the possible reasons for the limited transformation of these models to mobile devices. It is only hoped that future research might enable the implementation of these methods on mobile devices.

No studies were found on the transformation of these methods into clinical applications, which shows that their acceptance for clinical use is still limited. Many studies claim the value of their methods for use in clinical tasks; however, they lack reporting of testing for clinical purposes.

GANs were initially popular for generating synthetic image data similar to the original data. However, the perception of realistic-looking is subjective. Furthermore, though some quantitative measures such as peak signal-to-noise ratio (PSNR) and structural similarity index measure (SSIM) are reported in many included studies, these metrics are principally borrowed from the computer vision literature. Hence, how efficiently these metrics quantify the complex physiological information within the MRI images data is not well understood. Thus, there is a dire need to develop uniform methods to evaluate the performance of GANs on how well they capture complex features within MRI image data.

As used in many of the studies, the publicly available data for MRI are primarily from developed economies. However, there is a lack of medical imaging data from developing economies. Hence, computer models for diagnosis trained on such data may not necessarily generalize well for a population of different geoeconomics characteristics due to a lack of representation in the data. Including MRI data from diverse locations is needed and will help develop better AI methods for clinical applications such as diagnosis, prognosis, and tumor detection in brain MRI.

## Strengths and limitations

### Strengths

While many reviews have been published on the applications of GANs in medical imaging, to the best of our knowledge, this is the first review on the applications of GANs in brain MRI images. This review includes all the studies that used GANs for brain MRI; hence, this is the most comprehensive review on the topic. This review helps the readers to know the potential of the GANs for the synthesis of brain MRI data and the potential to improve the diagnosis and segmentation of brain tumors within brain MRI. Unlike reviews as [1–3] that covered a broad scope of different deep learning methods, this review focuses specifically on the applications of GANs in brain MRI.

In this review, we followed the scientific review guidelines of the PRISMA-ScR [4]. In addition, we covered the major databases in health sciences, engineering, and technology fields to identify as many as possible published studies. Hence, the number of studies included in this review is high. We devised a strategy to avoid bias in study selection by employing two independent reviewers for study selection and data extraction and a third reviewer to validate the screening and the data extraction. This review provides a comprehensive list of the publicly accessible datasets for brain MRI. Hence, it can be considered a rich resource for the readers to identify valuable datasets of brain MRI.

### Limitations

In this review, we included studies from five major databases. So, some studies might have been left out if they were not covered in the included databases. In addition, due to practical limitations, the review only consists of studies published in English. Hence, relevant studies published in other languages might have been left out. This review lists the studies into major applications such as synthesis, segmentation, diagnosis, super-resolution, and noise removal. The definition of some applications may overlap partly with others; for example, super-resolution may be considered as a sub-category of synthesis, and the categorization of super-resolution studies as synthesis studies will then increase the number of the studies in the synthesis category. However, we believe that the categorization in this review will better reflect the notion of the applications. We did not perform validation and assessment of the claims on the diagnosis of a brain tumor or the quality of the synthesized MRI data, as this was beyond the scope of this review.

## Conclusion

In this scoping review, we included 139 studies that reported the use of GANs for brain MRI data. We identified the most common applications of GANs. We also identified the most commonly used datasets publicly available for brain MRI. We also summarized the most common architectures of GANs and the evaluation metrics that are widely adopted to evaluate the performance of GANs in brain MRI. It will be most rewarding if these studies find their way into clinical transformations. To achieve this, we remark that encouraging the availability of the software and codes for these studies will facilitate the reproducibility of the results. Eventually, more research progress will be possible. In addition, the need to bridge the gap between the computer scientists and clinicians is widely felt as the input and feedback of clinicians and radiologists is vital for the research outcomes to find their way into clinical uses. Similarly, there is a need to follow standardized comparison protocols for the different architectures of GANs used for brain MRI.

## Supplementary Information

**Additional file1: Table S1** PRISMA-ScR Checklist. **Table S2** Search strategy. **Table S3** Interrater agreement matrices for study selection steps. **Table S4** Data extraction form. **Appendix S5** Characteristics of the studies.

## Data Availability

All data generated or analyzed during this study are included in this published article and its supplementary information files.

## Abbreviations

ACM: Association for Computing Machinery
BRaTs: Brain tumor segmentation
CAD: Computer-aided diagnosis
CT: Computed tomography
DSC: Dice similarity coefficient
FID: Fréchet inception distance
fMRI: Functional magnetic resonance imaging
GAN: Generative adversarial networks
IEEE: Institute of Electrical and Electronic Engineers
IXI: Information extraction from images
MAE: Mean absolute error
MRI: Magnetic resonance imaging
MSE: Mean square error
PRISMA-ScR: Preferred Reporting Items for Systematic Reviews and Meta-Analyses Extension for Scoping Reviews
PSNR: Peak signal-to-noise ratio
sMRI: Structural magnetic resonance imaging
SSIM: Structural similarity index measure
