# Correction: The role of generative adversarial networks in brain MRI: a scoping review

**DOI:** 10.1186/s13244-022-01268-7

**Published:** 2022-07-30

**Authors:** Hazrat Ali, Md. Rafiul Biswas, Farida Mohsen, Uzair Shah, Asma Alamgir, Osama Mousa, Zubair Shah

**Affiliations:** College of Science and Engineering, Hamad Bin Khalifa University, Qatar Foundation, 34110 Doha, Qatar

## Correction to: Insights into Imaging (2022) 13:98 10.1186/s13244-022-01237-0

The original article contained errors in Md. Rafiul Biswas's and Farida Mohsen's names which have since been corrected.

