# Supplementary material for: The role of generative adversarial networks in brain MRI: a scoping review
Source: Insights Imaging. 2022 Jun 4;13:98. doi: 10.1186/s13244-022-01237-0 (PMC9167371; doi:10.1186/s13244-022-01237-0)
Supplement: Supplementary file 1 — Additional file1: Table S1 PRISMA-ScR Checklist. Table S2 Search strategy. Table S3 Interrater agreement matrices for study selection steps. Table S4 Data extraction form. Appendix S5 Characteristics of the studies. [file 13244_2022_1237_MOESM1_ESM.docx]

**ELECTRONIC SUPPLEMENTARY MATERIAL**

**The Role of Generative Adversarial Networks in Brain MRI: A Scoping Review**

**Supplementary Table 1: PRISMA-ScR Checklist**

| SECTION | ITEM | PRISMA-ScR CHECKLIST ITEM | Reported in Section |
| --- | --- | --- | --- |
| TITLE | | | |
| Title | 1 | Identify the report as a scoping review. | Title page |
| ABSTRACT | | | |
| Structured summary | 2 | Provide a structured summary that includes (as applicable): background, objectives, methods, results, and conclusions that relate to the review questions and objectives. | Abstract |
| INTRODUCTION | | | |
| Rationale | 3 | Describe the rationale for the review in the context of what is already known. Explain why the review questions/objectives lend themselves to a scoping review approach. | Introduction |
| Objectives | 4 | Provide an explicit statement of the questions and objectives being addressed with reference to their key elements (e.g., population or participants, concepts, and context) or other relevant key elements used to conceptualize the review questions and/or objectives. | Introduction |
| METHODS | | | |
| Protocol and registration | 5 | Indicate whether a review protocol exists; state if and where it can be accessed (e.g., a Web address); and if available, provide registration information, including the registration number. | Methods |
| Eligibility criteria | 6 | Specify characteristics of the sources of evidence used as eligibility criteria (e.g., years considered, language, and publication status), and provide a rationale. | Methods |
| Information sources | 7 | Describe all information sources in the search (e.g., databases with dates of coverage and contact with authors to identify additional sources), as well as the date the most recent search was executed. | Methods |
| Search | 8 | Present the full electronic search strategy for at least 1 database, including any limits used, such that it could be repeated. | Methods and S2 Table |
| Selection of sources of evidence | 9 | State the process for selecting sources of evidence (i.e., screening and eligibility) included in the scoping review. | Methods |
| Data charting process | 10 | Describe the methods of charting data from the included sources of evidence (e.g., calibrated forms or forms that have been tested by the team before their use, and whether data charting was done independently or in duplicate) and any processes for obtaining and confirming data from investigators. | Methods and S4 Table |
| Data items | 11 | List and define all variables for which data were sought and any assumptions and simplifications made. | Methods |
| Critical appraisal of individual sources of evidence | 12 | If done, provide a rationale for conducting a critical appraisal of included sources of evidence; describe the methods used and how this information was used in any data synthesis (if appropriate). | N/A |
| Synthesis of results | 13 | Describe the methods of handling and summarizing the data that were charted. | Methods |
| RESULTS | | | |
| Selection of sources of evidence | 14 | Give numbers of sources of evidence screened, assessed for eligibility, and included in the review, with reasons for exclusions at each stage, ideally using a flow diagram. | Results |
| Characteristics of sources of evidence | 15 | For each source of evidence, present characteristics for which data were charted and provide the citations. | Results |
| Critical appraisal within sources of evidence | 16 | If done, present data on critical appraisal of included sources of evidence (see item 12). | N/A |
| Results of individual sources of evidence | 17 | For each included source of evidence, present the relevant data that were charted that relate to the review questions and objectives. | Results and S5 Table |
| Synthesis of results | 18 | Summarize and/or present the charting results as they relate to the review questions and objectives. | Results |
| DISCUSSION | | | |
| Summary of evidence | 19 | Summarize the main results (including an overview of concepts, themes, and types of evidence available), link to the review questions and objectives, and consider the relevance to key groups. | Discussion |
| Limitations | 20 | Discuss the limitations of the scoping review process. | Discussion |
| Conclusions | 21 | Provide a general interpretation of the results with respect to the review questions and objectives, as well as potential implications and/or next steps. | Conclusion |
| FUNDING | | | |
| Funding | 22 | Describe sources of funding for the included sources of evidence, as well as sources of funding for the scoping review. Describe the role of the funders of the scoping review. | N/A |

**Supplementary Table 2: Search Strategy**

Database(s): Pubmed, IEEE Xplore, ACM Digital Library, Scopus and Google Scholar.

Years: 2015 to 2022

Search dates: 20 September 2021 to 22 September 2021

Initial raw search results: 789

| **Database** | **Search strategy** | **Hits** |
| --- | --- | --- |
| IEEExplore | ((Generative Adversarial Networks) OR (GANs) OR (cycleGAN) OR (styleGAN)) AND ((Magnetic Resonance Imaging) OR (MRI) OR (fMRI) OR (sMRI)) AND (brain)) | 110 |
| ACM Digtial Library | [[Abstract: "generative adversarial networks"] OR [Abstract: "GANs"] OR [Abstract: "cycleGAN"] OR [Abstract: “styleGAN”]] AND [[Abstract: coronavirus] OR [Abstract: "covid-19"] OR [Abstract: COVID19] OR [Abstract: "sars-cov-2"] OR [Abstract: "2019-nCOV"] OR [Abstract: "corona pandemic"]] | 71 |
| Pubmed | (("Generative Adversarial Networks") OR ("GANs") OR ("cycleGAN") OR ("styleGAN")) AND (("Magnetic Resonance Imaging") OR ("MRI") OR ("fMRI") OR ("sMRI")) AND ("brain")) | 337 |
| Scopus | ( ( TITLE-ABS-KEY ( "Generative Adversarial Networks" ) OR TITLE-ABS-KEY ( "GANs" ) OR TITLE-ABS-KEY ( "cycleGAN" ) OR TITLE-ABS-KEY ( "styleGAN" ) ) ) AND ( ( TITLE-ABS-KEY ("Magnetic Resonance Imaging" ) ) OR ( TITLE-ABS-KEY ("MRI" ) ) OR ( TITLE-ABS-KEY ( "fMRI" ) ) OR ( TITLE-ABS-KEY ( "sMRI " ) ) AND ( ( TITLE-ABS-KEY ( "brain" ) ) ) AND ( LIMIT-TO ( PUBYEAR , 2021 ) OR LIMIT-TO ( PUBYEAR , 2015 ) ) | 171 |
| Google Scholar | ((Generative Adversarial Networks) OR (GANs) OR (cycleGAN) OR (styleGAN)) AND ((Magnetic Resonance Imaging) OR (MRI) OR (fMRI) OR (sMRI)) AND (brain)) | 100 |

**Supplementary Table 3: Interrater agreement matrices for study selection steps.**

**Title and abstract screening**

|  | | **Reviewer 1 (MB)** | | |
| --- | --- | --- | --- | --- |
|  |  | **Include** | **Exclude** | **Total** |
| **Reviewer 2 (FA)** | **Include** | **142** | **18** | **160** |
|  | **Exclude** | **14** | **430** | **444** |
|  | **Total** | **156** | **448** | **604** |

Cohen Kappa = 0.863

**Full text reading**

|  | | **Reviewer 1 (MB)** | | |
| --- | --- | --- | --- | --- |
|  |  | **Include** | **Exclude** | **Total** |
| **Reviewer 2 (FA)** | **Include** | **137** | **3** | **140** |
|  | **Exclude** | **2** | **16** | **18** |
|  | **Total** | **139** | **19** | **158** |

Cohen Kappa = 0.847

**Supplementary Table 4: Data extraction form**

| **Concept** | **Definition** |
| --- | --- |
| **Study Characteristics** |  |
| ID | Unique ID assigned to each study |
| Author | The first author of the study. |
| Year | The year in which the study was published |
| Country of publication | Affiliation of the first author of the study. |
| Publication type | Journal or conference or book chapter |
| Conference name | Name of the conference where the study was published |
| Journal name | Name of the journal where the study was published |
| **GANs method** |  |
| Tasks addressed in the study | What are the applications addressed in the study (e.g., synthesis, diagnosis, prognosis)? |
| Purpose of using GAN | What was the specific purpose of using GAN (e.g., for data generation, segmentation, noise removal, segmenation, etc)? |
| Type of GAN | What was the architecture of GAN that was used (e.g., cycleGAN, conditional GAN, Deep Convolutional GAN)? |
| Key changes in GAN | Did the authors report fundamental changes to the architecture of the GAN? |
|  |  |
| **Dataset** **Characteristics** |  |
| Type of data sources | Public or private |
| Name of the data source if available | The specific name of the dataset or the source (for example, github or Kaggle) |
| Data source (full URL) | Mention full URL of the dataset |
| Dataset size (Number of subjects or individuals) | For how many individuals the data is recorded? |
| Dataset size (number of images) | What is the total number of images in the dataset? |
| Training set | What is the number of images in the training set? |
| Validation set size | What is the number of images in the validation set? |
| Testing set size | What is the number of images in the test set? |
| **Evaluation** |  |
| Type of validation | What type of validation the authors used? (e.g., train test split, k-fold cross validation, external validation) |
| Evaluation metrics | Metrics of evaluation (for example, Accuracy, Precision, Recall, Dice socre, F1 score, Sensitivitiy, Specificity, SSIM, PSNR) |
| Study aim | One statement summary of the work. |

**Appendix 5: Characteristics of the studies.**

| ID | First Author Name | Title | Year | Publication Type | Conference | Journal | Country | Application of study | Type of dataset |
| --- | --- | --- | --- | --- | --- | --- | --- | --- | --- |
| S1 | Changhee | Combining Noise-to-Image and Image-to-Image GANs Brain MR Image Augmentation for Tumor Detection | 2019 | journal | N/A | IEEE Access | Japan | synthesis | public |
| S2 | \| Xiaofeng \| \| --- \| | Multimodal MRI synthesis using unified generative adversarial networks | 2020 | journal | N/A | Medical Physics | USA | synthesis | public |
| S3 | Anmol | Missing MRI Pulse Sequence Synthesis Using Multi-Modal Generative Adversarial Network | 2020 | journal | N/A | IEEE TRANSACTIONS ON MEDICAL IMAGING | Canada | synthesis | public |
| S4 | Yawen Huang | MCMT-GAN: Multi-Task Coherent Modality Transferable GAN for 3D Brain Image Synthesis | 2020 | journal | N/A | IEEE TRANSACTIONS ON IMAGE PROCESSING | USA | synthesis | public |
| S5 | Bingyu | MULTI-MODALITY GENERATIVE ADVERSARIAL NETWORKS WITH TUMOR CONSISTENCY LOSS FOR BRAIN MR IMAGE SYNTHESIS | 2020 | conference | International Symposium on Biomedical Imaging (ISBI) | N/A | China | synthesis | public |
| S6 | Xiaoming Liu | Multimodal MR Image Synthesis Using Gradient Prior and Adversarial Learning | 2020 | journal | N/A | IEEE JOURNAL OF SELECTED TOPICS IN SIGNAL PROCESSING | China | synthesis | public |
| S7 | Chee | Synthesis of 3D MRI Brain Images With Shape and Texture Generative Adversarial Deep Neural Networks | 2021 | journal | N/A | IEEE Acces | Malaysia | synthesis | public |
| S8 | Xin | Bi-Modality Medical Image Synthesis Using Semi-Supervised Sequential Generative Adversarial Networks | 2020 | journal | N/A | IEEE JOURNAL OF BIOMEDICAL AND HEALTH INFORMATICS | China | synthesis | public |
| S9 | Changhee | GAN-BASED SYNTHETIC BRAIN MR IMAGE GENERATION | 2018 | conference | International Symposium on Biomedical Imaging (ISBI 2018) | N/A | Japan | synthesis | public |
| S10 | \| Yili Qu \| \| --- \| | Multimodal Brain MRI Translation Focused on Lesions | 2020 | journal | N/A | ACM | China | synthesis | public |
| S11 | Emanuel Alogna | Brain Magnetic Resonance Imaging Generation using Generative Adversarial Networks | 2020 | conference | 2020 IEEE Symposium Series on Computational Intelligence (SSCI) | N/A | Italy | synthesis | public |
| S12 | \| Yan Li \| \| --- \| | Synthesize CT from paired MRI of the same patient with patch-based generative adversarial network | 2021 | conference | Progr. Biomed. Opt. Imaging Proc. SPIE | N/A | China | synthesis | private |
| S13 | Yandi Gu | AIDS Brain MRIs Synthesis via Generative Adversarial Networks Based on Attention-Encoder | 2020 | conference | 2020 IEEE 6th International Conference on Computer and Communications | N/A | China | synthesis | public, private |
| S14 | R. T. R | Artificial MRI Image Generation using Deep Convolutional GAN and its Comparison with other Augmentation Methods | 2021 | conference | 2021 International Conference on Communication, Control and Information Sciences (ICCISc) | N/A | India | synthesis | public |
| S15 | Mina Rezaei | Generative synthetic adversarial network for internal bias correction and handling class imbalance problem in medical image diagnosis | 2020 | conference | Progr. Biomed. Opt. Imaging Proc. SPIE | N/A | Germany | synthesis | private |
| S16 | Dikici E. | Constrained generative adversarial network ensembles for sharable synthetic medical images | 2021 | journal | N/A | Journal of Medical Imaging | USA | synthesis | public |
| S17 | Sunho Kim | Synthesis of brain tumor multicontrast MR images for improved data augmentation. | 2021 | journal | N/A | Medical physics | South Korea | synthesis | public |
| S18 | Haider Al-Tahan | Reconstructing feedback representations in the ventral visual pathway with a generative adversarial autoencoder | 2021 | journal | N/A | PLoS computational biology | Canada | synthesis | public |
| S19 | Li, D. | Multi-subject data augmentation for target subject semantic decoding with deep multi-view adversarial learning | 2021 | journal | N/A | Information Sciences | China | synthesis | public |
| S20 | \| BaoqiangMa \| \| --- \| | MRI image synthesis with dual discriminator adversarial learning and difficulty-aware attention mechanism for hippocampal subfields segmentation | 2020 | journal | N/A | Computerized Medical Imaging and Graphics | China | synthesis | public |
| S21 | Shan Zhang | An Auto-Encoding Generative Adversarial Networks for Generating Brain Network | 2020 | conference | ACM | N/A | China | synthesis | public |
| S22 | Alice | Data Augmentation of 3D Brain Environment Using Deep Convolutional Refined Auto-Encoding Alpha GAN | 2021 | journal | N/A | IEEE TRANSACTIONS ON MEDICAL ROBOTICS AND BIONICS | Italy | synthesis | public |
| S23 | Huan Yang | An Indirect Multimodal Image Registration and Completion Method Guided by Image Synthesis | 2020 | journal | N/A | Computational and Mathematical Methods in Medicine | China | synthesis | private |
| S24 | Samaneh Kazemifar | MRI-only brain radiotherapy: Assessing the dosimetric accuracy of synthetic CT images generated using a deep learning approach | 2019 | journal | N/A | Radiotherapy and Oncology | USA | synthesis | private |
| S25 | Berardino | Data augmentation using generative adversarial neural networks on brain structural connectivity in multiple sclerosis. | 2021 | journal | N/A | Computer methods and programs in biomedicine | Ireland | synthesis | private |
| S26 | Alejandro | Realistic generation of diffusion-weighted magnetic resonance brain images with deep generative models. | 2021 | journal | N/A | Magnetic resonance imaging | Netherlands | synthesis | private |
| S27 | Jeremiah | Synthetic generation of DSC-MRI-derived relative CBV maps from DCE MRI of brain tumors. | 2021 | journal | N/A | Magnetic resonance in medicine | USA | synthesis | private |
| S28 | Tom Finck | Deep-Learning Generated Synthetic Double Inversion Recovery Images Improve Multiple Sclerosis Lesion Detection | 2020 | journal | N/A | Investigative Radiology | Germany | synthesis | private |
| S29 | Koshino Kazuhiro | Generative Adversarial Networks for the Creation of Realistic Artificial Brain Magnetic Resonance Images | 2018 | journal | N/A | tomography | Japan | synthesis | private |
| S30 | \| Deepak \| \| --- \| | MSG-GAN Based Synthesis of Brain MRI with Meningioma for Data Augmentation | 2020 | conference | 2020 IEEE International Conference on Electronics, Computing and Communication Technologies (CONECCT) | N/A | India | synthesis | public |
| S31 | GUANGYUAN | A Modified Generative Adversarial Network Using Spatial and Channel-Wise Attention for CS-MRI Reconstruction | 2021 | journal | N/A | IEEE Access | China | synthesis | public |
| S32 | Chenjie | CROSS-MODALITY AUGMENTATION OF BRAIN MR IMAGES USING A NOVEL PAIRWISE GENERATIVE ADVERSARIAL NETWORK FOR ENHANCED GLIOMA CLASSIFICATION | 2019 | conference | ICIP | N/A | Sweden | synthesis | public |
| S33 | Zhang Hongtao | 3D Brain MRI Reconstruction based on 2D Super-Resolution Technology | 2020 | conference | 2020 IEEE International Conference on Systems, Man, and Cybernetics (SMC | N/A | Japan | 3D synthesis | private |
| S34 | X. Zhang | Deep Neural Networks with Broad Views for Parkinson's Disease Screening | 2019 | conference | 2019 IEEE International Conference on Bioinformatics and Biomedicine (BIBM) | N/A | China | synthesis | public |
| S35 | Kai Qiao | BigGAN-based Bayesian Reconstruction of Natural Images from Human Brain Activity | 2020 | journal | N/A | Neuroscience | China | synthesis | public |
| S36 | Yuhei Koike | Feasibility of synthetic computed tomography generated with an adversarial network for multi-sequence magnetic resonance-based brain radiotherapy | 2019 | journal | N/A | Journal of Radiation Research | Japan | synthesis | public |
| S37 | Shengye | Bidirectional Mapping Generative Adversarial Networks for Brain MR to PET Synthesis. | 2021 | journal | N/A | IEEE transactions on medical imaging | USA | synthesis | public |
| S38 | AlaaAbu-Srhan | Paired-unpaired Unsupervised Attention Guided GAN with transfer learning for bidirectional brain MR-CT synthesis | 2021 | journal | N/A | Computers in Biology and Medicine | Jordan | synthesis | public, private |
| S39 | GM Conte | Generative Adversarial Networks to Synthesize Missing T1 and FLAIR MRI Sequences for Use in a Multisequence Brain Tumor Segmentation Model. | 2021 | journal | N/A | Radiology | USA | synthesis | private |
| S40 | Francesco | MPRAGE to MP2RAGE UNI translation via generative adversarial network improves the automatic tissue and lesion segmentation in multiple sclerosis patients. | 2021 | journal | N/A | Computers in biology and medicine | USA | synthesis | private |
| S41 | Tang | Dosimetric evaluation of synthetic CT image generated using a neural network for MR-only brain radiotherapy. | 2021 | journal | N/A | The Journal of Applied Clinical Medical Physics (JACMP) | China | synthesis | private |
| S42 | Gu Y. | A Transfer Deep Generative Adversarial Network Model to Synthetic Brain CT Generation from MR Images | 2021 | journal | N/A | Wireless Communications and Mobile Computing | China | synthesis | public |
| S43 | Dapeng | Research on the Modality Transfer Method of Brain Imaging Based on Generative Adversarial Network. | 2021 | journal | N/A | Frontiers in neuroscience | China | synthesis | private |
| S44 | Yang Lei | Multi-modality MRI arbitrary transformation using unified generative adversarial networks | 2020 | conference | Proceedings SPIE | N/A | USA | synthesis | private |
| S45 | HAO | Brain Tumor Segmentation with Generative Adversarial Nets | 2019 | conference | IEEE International Conference on Artificial Intelligence and Big Data | N/A | China | segmentation | public |
| S46 | \| Yuta \| \| --- \| | An Inductive Transfer Learning Approach using Cycle-consistent Adversarial Domain Adaptation with Application to Brain Tumor | 2019 | conference | Proceedings of the 2019 6th International Conference on Biomedical and Bioinformatics Engineering | N/A | Japan | segmentation | public |
| S47 | Hosna | Data Efficient Segmentation of various 3D Medical Images using Guided Generative Adversarial Networks | 2020 | journal | N/A | IEEE Access | South Korea | segmentation | public, private |
| S48 | Biting | 3D CGAN BASED CROSS-MODALITY MR IMAGE SYNTHESIS FOR BRAIN TUMOR SEGMENTATION | 2018 | conference | International Symposium on Biomedical Imaging (ISBI 2018) | N/A | Australia | segmentation | public |
| S49 | W. Wu | Deep Learning for Neuroimaging Segmentation with a Novel Data Augmentation Strategy | 2020 | conference | 2020 42nd Annual International Conference of the IEEE Engineering in Medicine & Biology Society (EMBC) | N/A | Singapore | segmentation | public |
| S50 | Mohammad Hamghalam | High tissue contrast image synthesis via multistage attention-GAN: Application to segmenting brain MR scans. | 2020 | journal | N/A | Neural networks | China | segmentation | public |
| S51 | Mohammad Hamghalam | High tissue contrast MRI synthesis using multi-stage attention-GAN for glioma segmentation | 2020 | conference | AAAI Conference on Artificial Intelligence | N/A | China | segmentation | public |
| S52 | Hyunhee Lee | Study on Optimal Generative Network for Synthesizing Brain Tumor-Segmented MR Images | 2020 | journal | N/A | Mathematical Problems in Engineering | South Korea | segmentation | private |
| S53 | Carver | Improvement of Multiparametric MR Image Segmentation by Augmenting the Data With Generative Adversarial Networks for Glioma Patients | 2021 | journal | N/A | Frontiers in Computational Neuroscience | USA | segmentation | public |
| S54 | Tabea Kossen | Synthesizing anonymized and labeled TOF-MRA patches for brain vessel segmentation using generative adversarial networks. | 2021 | journal | N/A | Computers in biology and medicine | Germany | segmentation | private |
| S55 | Yue Chen | Efficient_3D_Neural_Networks_with_Support_Vector_Machine_for_Hippocampus_Segmentation | 2020 | conference | International Conference on Artificial Intelligence and Computer Engineering | N/A | China | segmentation | private |
| S56 | \| Hao Chen \| \| --- \| | Brain Tumor Segmentation with Generative Adversarial Nets | 2019 | conference | 2019 2nd International Conference on Artificial Intelligence and Big DataC | N/A | China | segmentation | public |
| S57 | Mohammad Hamghalam | TRANSFORMING INTENSITY DISTRIBUTION OF BRAIN LESIONS VIA CONDITIONAL GANS FOR SEGMENTATION | 2020 | conference | 2020 IEEE 17th International Symposium on Biomedical Imaging (ISBI) | N/A | China | segmentation | public |
| S58 | Nan Xi | Semi-supervised Attentive Mutual-info Generative Adversarial Network for Brain Tumor Segmentation | 2019 | conference | N/A | N/A | China | segmentation | public |
| S59 | Thirumagal | Design of FCSE-GAN for Dissection of Brain Tumour in MRI | 2020 | conference | International Conference on Smart Technologies in Computing, Electrical and Electronics (ICSTCEE) | N/A | India | segmentation | public |
| S60 | P. Zhuang | Synthetic Power Analyses: Empirical Evaluation and Application to Cognitive Neuroimaging | 2019 | conference | 2019 53rd Asilomar Conference on Signals, Systems, and Computers | N/A | USA | synthesis | public |
| S61 | Yili Qu | Multimodal Brain MRI Translation Focused on Lesions | 2020 | conference | ICMLC 2020 | N/A | China | segmentation | public |
| S62 | H. Asma-Ull | Data Efficient Segmentation of Various 3D Medical Images Using Guided Generative Adversarial Networks | 2020 | journal | N/A | IEEE Access | South Korea | segmentation | public |
| S63 | J. Das | Brain Tumor Segmentation Using Discriminator Loss | 2019 | conference | 2019 National Conference on Communications (NCC) | N/A | India | segmentation | public |
| S64 | Jose Bernal | Generating Longitudinal Atrophy Evaluation Datasets on Brain Magnetic Resonance Images Using Convolutional Neural Networks and Segmentation Priors | 2021 | journal | N/A | Neuroinformatics | Spain | segmentation | public |
| S65 | Z. Tang | Multi-Atlas Brain Parcellation Using Squeeze-and-Excitation Fully Convolutional Networks | 2020 | journal | N/A | IEEE Transactions on Image Processing | China | segmentation | public |
| S66 | M. Rezaei | Conditional Generative Adversarial Refinement Networks for Unbalanced Medical Image Semantic Segmentation | 2019 | conference | 2019 IEEE Winter Conference on Applications of Computer Vision (WACV) | N/A | Germany | segmentation | public |
| S67 | \| Li Tao \| \| --- \| | Pseudo CT Image Synthesis and Bone Segmentation From MR Images Using Adversarial Networks With Residual Blocks for MR-Based Attenuation Correction of Brain PET Data | 2021 | journal | N/A | IEEE TRANSACTIONS ON RADIATION AND PLASMA MEDICAL SCIENCES, | China | segmentation | public, private |
| S68 | D. Mahapatra | Training Data Independent Image Registration with Gans Using Transfer Learning and Segmentation Information | 2019 | conference | 2019 IEEE 16th International Symposium on Biomedical Imaging (ISBI 2019) | N/A | Australia | segmentation | public |
| S69 | Y. Hou | Brain Tumor Segmentation based on Knowledge Distillation and Adversarial Training | 2021 | conference | 2021 International Joint Conference on Neural Networks (IJCNN) | N/A | China | segmentation | public |
| S70 | Xinheng Wu | Unsupervised brain tumor segmentation using a symmetric-driven adversarial network | 2021 | journal | N/A | Neurocomputing | Australia | segmentation | public |
| S71 | Cheng G. | Correcting and reweighting false label masks in brain tumor segmentation | 2021 | journal | N/A | Medical Physics | China | segmentation | public |
| S72 | Wenguang Yuan | Unified generative adversarial networks for multimodal segmentation from unpaired 3D medical images | 2020 | journal | N/A | Medical Image Analysis | China | segmentation | public |
| S73 | Jing Liu | Glioma subregions segmentation with a discriminative adversarial regularized 3D unet | 2019 | conference | ACM Int. Conf. Proc. Ser. | N/A | China | segmentation | public |
| S74 | Yilin Liu | A 3D Fully Convolutional Neural Network With Top-Down Attention-Guided Refinement for Accurate and Robust Automatic Segmentation of Amygdala and Its Subnuclei | 2020 | journal | N/A | Frontiers in Neuroscience | USA | segmentation | private |
| S75 | Yonggang Shi | Hippocampal subfields segmentation in brain MR images using generative adversarial networks | 2019 | journal | N/A | BioMedical Engineering OnLine | China | segmentation | private |
| S76 | Nuo Tong | Shape constrained fully convolutional DenseNet with adversarial training for multiorgan segmentation on head and neck CT and low-field MR images | 2019 | journal | N/A | Medical Physics | USA | segmentation | private |
| S77 | Jiayin | Fusion of Brain PET and MRI Images Using Tissue-Aware Conditional Generative Adversarial Network With Joint Loss | 2020 | journal | N/A | IEEE Access | China | diagnosis | public |
| S78 | Chenjie | Enlarged Training Dataset by Pairwise GANs for Molecular-Based Brain Tumor Classification | 2020 | journal | N/A | IEEE Access | Sweden | diagnosis | public |
| S79 | Wanyun Lin | Synthesizing Missing Data using 3D Reversible GAN for Alzheimer’s Disease | 2020 | conference | 2020 Association for Computing Machinery | N/A | China | diagnosis | public |
| S80 | \| Yongsheng Pan \| \| --- \| | Spatially-Constrained Fisher Representation for Brain Disease Identification With Incomplete Multi-Modal Neuroimages | 2020 | journal | N/A | IEEE TRANSACTIONS ON MEDICAL IMAGING, | China | diagnosis | public |
| S81 | T. Chen | Preserving-Texture Generative Adversarial Networks for Fast Multi-Weighted MRI | 2018 | journal | N/A | IEEE Access | China | diagnosis | public |
| S82 | Wen Yu | Tensorizing GAN With High-Order Pooling for Alzheimer’s Disease Assessment | 2021 | journal | N/A | IEEE Transactions on Neural Networks and Learning Systems | China | diagnosis | public |
| S83 | Zhou, X | Enhancing magnetic resonance imaging-driven Alzheimer’s disease classification performance using generative adversarial learning | 2021 | journal | N/A | Alzheimer's Research and Therapy | USA | diagnosis | public |
| S84 | \| Dinesh \| \| --- \| | Brain Tumor Classification based on MR Images using GAN as a Pre-Trained Model | 2021 | conference | 2021 IEEE Ural-Siberian Conference on Computational Technologies in Cognitive Science, Genomics and Biomedicine (CSGB) | N/A | Russia | diagnosis | public |
| S85 | Navid Ghassemi | Deep neural network with generative adversarial networks pre-training for brain tumor classification based on MR images | 2019 | journal | N/A | Biomedical Signal Processing and Control | Iran | diagnosis | public |
| S86 | Teguh | Dual-encoder Bidirectional Generative Adversarial Networks for Anomaly Detection | 2020 | conference | 19th IEEE International Conference on Machine Learning and Applications (ICMLA) | N/A | Japan | diagnosis | public |
| S87 | Xingyu | Task-induced Pyramid and Attention GAN for Multimodal Brain Image Imputation and Classification in Alzheimers disease. | 2021 | journal | N/A | IEEE journal of biomedical and health informatics | USA | diagnosis | public |
| S88 | B. Sandhiya | Reconstruction, Identification and Classification of Brain Tumor Using Gan and Faster Regional-CNN | 2021 | conference | 2021 3rd International Conference on Signal Processing and Communication (ICPSC) | N/A | India | diagnosis | private |
| S89 | Changhee Han | MADGAN: unsupervised medical anomaly detection GAN using multiple adjacent brain MRI slice reconstruction. | 2021 | conference | Computational Intelligence methods for Bioinformatics and Biostatistics Bergamo, Italy. 4-6 September 2019 | BMC bioinformatics | Japan | diagnosis | private |
| S90 | Wanyun | Bidirectional Mapping of Brain MRI and PET With 3D Reversible GAN for the Diagnosis of Alzheimer's Disease. | 2021 | journal | N/A | Frontiers in neuroscience | China | diagnosis | public |
| S91 | Da Ma | Differential Diagnosis of Frontotemporal Dementia, Alzheimer’s Disease, and Normal Aging Using a Multi-Scale Multi-TypeFeature Generative Adversarial Deep Neural Network on Structural Magnetic Resonance Images | 2020 | journal | N/A | Frontiers in Neuroscience | China | diagnosis | public |
| S92 | Kaur S. | Diagnosis of Parkinson’s disease using deep CNN with transfer learning and data augmentation | 2021 | journal | N/A | Multimedia Tools and Applications | India | diagnosis | public |
| S93 | Chenjie Ge | Deep semi-supervised learning for brain tumor classification | 2020 | journal | N/A | BMC medical imaging | Sweden | diagnosis | public |
| S94 | Changhee Han | Learning more with less: Conditional PGGAN-based data augmentation for brain metastases detection using highly-rough annotation on MR images | 2019 | conference | Int Conf Inf Knowledge Manage | N/A | China | diagnosis | private |
| S95 | Samaneh Kazemifar | Dosimetric evaluation of synthetic CT generated with GANs for MRI-only proton therapy treatment planning of brain tumors. | 2020 | journal | N/A | Journal of Applied Clinical Medical Physics | USA | diagnosis | private |
| S96 | \| Mina Rezaei \| \| --- \| | Generative Adversarial Framework for Learning Multiple Clinical Tasks | 2018 | conference | N/A | N/A | Germany | diagnosis | public |
| S97 | Bao | UNSUPERVISED REGION-BASED ANOMALY DETECTION IN BRAIN MRI WITH ADVERSARIAL IMAGE INPAINTING | 2021 | conference | International Symposium on Biomedical Imaging (ISBI) | N/A | UK | diagnosis | public |
| S98 | Datta. s. | A deep framework for enhancement of diagnostic information in CSMRI reconstruction | 2022 | journal | N/A | Biomedical Signal Processing and Control | India | diagnosis | public |
| S99 | C. H Pham | SIMULTANEOUS SUPER-RESOLUTION AND SEGMENTATION USING A GENERATIVE ADVERSARIAL NETWORK: APPLICATION TO NEONATAL BRAIN MRI ADVERSARIAL NETWORK: APPLICATION TO NEONATAL BRAIN MRI | 2019 | conference | International Symposium on Biomedical Imaging (ISBI 2019) | N/A | France | super resolution | public |
| S100 | Quentin | SegSRGAN: Super-resolution and segmentation using generative adversarial networks — Application to neonatal brain MRI | 2020 | journal | N/A | Computers in biology and Medicine | France | super resolution | public |
| S101 | TIAO | Preserving-Texture Generative Adversarial Networks for Fast Multi-Weighted MRI | 2017 | journal | N/A | IEEE Access | China | super resolution | public |
| S102 | Vincent | Dosimetric Validation of a GAN-Based Pseudo-CT Generation for MRI-Only Stereotactic Brain Radiotherapy. | 2020 | journal | N/A | Cancers | France | super resolution | public |
| S103 | Hongtao Zhang | 3D MRI Reconstruction Based on 2D Generative Adversarial Network Super-Resolution | 2021 | journal | N/A | Sensors (Basel, Switzerland) | Japan | super resolution | public |
| S104 | Jin | Arbitrary Scale Super-Resolution for Medical Images. | 2021 | journal | N/A | International journal of neural systems | Singapore | super resolution | public |
| S105 | Yawen Huang | Super-resolution and inpainting with degraded and upgraded generative adversarial networks | 2020 | conference | IJCAI-20: International Joint Conference on Artificial Intelligence | N/A | China | super resolution | public |
| S106 | Shuo | SLICE PROFILE ESTIMATION FROM 2D MRI ACQUISITION USING GENERATIVE ADVERSARIAL NETWORKS | 2021 | conference | 18th International Symposium on Biomedical Imaging (ISBI) | N/A | USA | super resolution | private |
| S107 | Yuchong Gu | MedSRGAN: medical images super-resolution using generative adversarial networks | 2020 | journal | N/A | Multimedia Tools and Applications | China | super resolution | private |
| S108 | Tal | DEEP SEMI-SUPERVISED BIAS FIELD CORRECTION OF MR IMAGES | 2021 | conference | 18th International Symposium on Biomedical Imaging (ISBI) | N/A | Israel | noise removal | public |
| S109 | Hossein | Novel adversarial semantic structure deep learning for MRI-guided attenuation correction in brain PET/MRI | 2019 | journal | N/A | European journal of nuclear medicine and molecular imaging | Germany | noise removal | public |
| S110 | Patricia M. Johson | Conditional generative adversarial network for 3D rigid-body motion correction in MRI | 2019 | journal | N/A | Magnetic resonance in medicine | Canada | noise removal | public |
| S111 | Maosong Ran | Denoising of 3D magnetic resonance images using a residual encoder-decoder Wasserstein generative adversarial network. | 2019 | journal | N/A | Medical Image Analysis | China | noise removal | public |
| S112 | K. Armanious | ipA-MedGAN: Inpainting of Arbitrary Regions in Medical Imaging | 2020 | conference | 2020 IEEE International Conference on Image Processing (ICIP) | N/A | Germany | noise removal | private |
| S113 | Akifumi | Improving the Quality of Synthetic FLAIR Images with Deep Learning Using a Conditional Generative Adversarial Network for Pixel-by-Pixel Image Translation. | 2019 | journal | N/A | AJNR. American journal of neuroradiology | Japan | super resolution | private |
| S114 | \| H Zhang \| \| --- \| | Reconstructing the Perceived Faces from Brain Signals without Large Number of Training Samples | 2020 | conference | IEEE International Conference | N/A | China | reconstruction | public |
| S115 | Hajar Emami | Attention-Guided Generative Adversarial Network to Address Atypical Anatomy in Synthetic CT Generation | 2020 | conference | 2020 IEEE 21st International Conference on Information Reuse and Integration for Data Science (IRI) | N/A | USA | reconstruction | public, private |
| S116 | Kai Wang | Compressed Sensing MRI Reconstruction using GAN with Enhanced Antagonism | 2019 | conference | 2019 12th International Conference on Intelligent Computation Technology and Automation (ICICTA) | N/A | China | reconstruction | public |
| S117 | Milad Mozafari | Reconstructing Natural Scenes from fMRI Patterns using BigBiGAN | 2020 | conference | N/A | N/A | France | reconstruction | public |
| S118 | Salman U.H. Dar | Prior-Guided Image Reconstruction for Accelerated Multi-Contrast MRI via Generative Adversial Networks | 2020 | journal | N/A | IEEE JOURNAL OF SELECTED TOPICS IN SIGNAL PROCESSING | Turkey | reconstruction | public |
| S119 | G. Li | A Modified Generative Adversarial Network Using Spatial and Channel-Wise Attention for CS-MRI Reconstruction | 2021 | journal | N/A | IEEE Access | China | reconstruction | public |
| S120 | Roy Shaul | Subsampled brain MRI reconstruction by generative adversarial neural networks | 2020 | journal | N/A | Medical image analysis | Israel | reconstruction | public |
| S121 | Ziqi Ren | Reconstructing seen image from brain activity by visually-guided cognitive representation and adversarial learning. | 2021 | journal | N/A | NeuroImage | Israel | reconstruction | public |
| S122 | Guohua Shen | End-to-end deep image reconstruction from human brain activity | 2019 | journal | N/A | Frontiers in Computational Neuroscience | Japan | reconstruction | public |
| S123 | Jun Lv | Transfer learning enhanced generative adversarial networks for multi-channel MRI reconstruction | 2021 | journal | N/A | Computers in Biology and Medicine | China | reconstruction | public, private |
| S124 | \| JIAQI GU \| \| --- \| | Deep Generative Adversarial Networks for Thin-Section Infant MR Image Reconstruction | 2019 | journal | N/A | IEEE Access | China | reconstruction | private |
| S125 | Won Joon Do | Reconstruction of multiconstrast MR images through deep learning | 2020 | journal | N/A | Medical physics | South Korea | reconstruction | private |
| S126 | Yicheng Chen | QSMGAN: Improved Quantitative Susceptibility Mapping using 3D Generative Adversarial Networks with increased receptive field. | 2020 | journal | N/A | NeuroImage | USA | reconstruction | private |
| S127 | Viktor | GENERATIVE AGING OF BRAIN MRI FOR EARLY PREDICTION OF MCI-AD CONVERSION | 2019 | conference | 16th International Symposium on Biomedical Imaging (ISBI 2019) | N/A | Switzerland | prediction | public |
| S128 | Sunho | Domain Mapping and Deep Learning from Multiple MRI Clinical Datasets for Prediction of Molecular Subtypes in Low Grade Gliomas. | 2020 | journal | N/A | Medical Physics | South Korea | prediction | private |
| S129 | Alaa | Brain multigraph prediction using topology-aware adversarial graph neural network. | 2021 | journal | N/A | Medical image analysis | Netherlands | prediction | public |
| S130 | Junzhong | Estimating Effective Connectivity by Recurrent Generative Adversarial Networks. | 2021 | journal | N/A | IEEE transactions on medical imaging | USA | prediction | public |
| S131 | \| Ahmad \| \| --- \| | Glioma Growth Prediction via Generative Adversarial Learning from Multi-Time Points Magnetic Resonance Images | 2020 | conference | International Conference of the IEEE Engineering in Medicine & Biology Society (EMBC), 2020, | N/A | China | prediction | private |
| S132 | Wen | Predicting PET-derived myelin content from multisequence MRI for individual longitudinal analysis in multiple sclerosis. | 2020 | journal | N/A | NeuroImage | USA | prediction | private |
| S133 | Jinduo Liu | EC-GAN: Inferring brain effective connectivity via generative adversarial networks | 2020 | conference | AAAI Conference on Artificial Intelligence | N/A | China | prediction | public |
| S134 | Yan Zhao | Prediction of Alzheimer’s Disease Progression with Multi-Information Generative Adversarial Network | 2020 | journal | N/A | IEEE JOURNAL OF BIOMEDICAL AND HEALTH INFORMATICS | China | prognosis | public |
| S135 | S. Roychowdhury | A Modular Framework to Predict Alzheimerâ€™s Disease Progression Using Conditional Generative Adversarial Networks | 2020 | conference | 2020 International Joint Conference on Neural Networks (IJCNN) | N/A | USA | prognosis | public |
| S136 | Muhammad Febrian Rachmadi | Predicting the evolution of white matter Hyperintensities in brain MRI using generative adversarial networks and irregularity map | 2019 | conference | International Conference on Medical Image Computing and Computer-Assisted Intervention |  | UK | prognosis | public |
| S137 | Ahmed Elazab | GP-GAN: Brain tumor growth prediction using stacked 3D generative adversarial networks from longitudinal MR Images | 2020 | journal | N/A | Neural Networks | China | prognosis | public, private |
| S138 | Dwarikanath Mahapatra | Training data independent image registration using generative adversarial networks and domain adaptation | 2020 | journal | N/A | Pattern Recognition | United Arab Emirates | image registration | public |
| S139 | Yuanjie Zheng | SymReg-GAN: Symmetric Image Registration with Generative Adversarial Networks | 2021 | journal | N/A | IEEE Transactions on Pattern Analysis and Machine Intelligence 1 | China | image registration | public |
